# A Double-Edged Sword: How Oncogenes and Tumor Suppressor Genes Can Contribute to Chromosomal Instability

**DOI:** 10.3389/fonc.2013.00164

**Published:** 2013-06-27

**Authors:** Bernardo Orr, Duane A. Compton

**Affiliations:** ^1^Department of Biochemistry, Geisel School of Medicine at Dartmouth, Hanover, NH, USA; ^2^The Norris-Cotton Cancer Center, Geisel School of Medicine at Dartmouth, Hanover, NH, USA

**Keywords:** aneuploidy, chromosomal instability, CIN, oncogenic signaling, mitosis, chromosome segregation, cancer, genomic instability

## Abstract

Most solid tumors are characterized by abnormal chromosome numbers (aneuploidy) and karyotypic profiling has shown that the majority of these tumors are heterogeneous and chromosomally unstable. Chromosomal instability (CIN) is defined as persistent mis-segregation of whole chromosomes and is caused by defects during mitosis. Large-scale genome sequencing has failed to reveal frequent mutations of genes encoding proteins involved in mitosis. On the contrary, sequencing has revealed that most mutated genes in cancer fall into a limited number of core oncogenic signaling pathways that regulate the cell cycle, cell growth, and apoptosis. This led to the notion that the induction of oncogenic signaling is a separate event from the loss of mitotic fidelity, but a growing body of evidence suggests that oncogenic signaling can deregulate cell cycle progression, growth, and differentiation as well as cause CIN. These new results indicate that the induction of CIN can no longer be considered separately from the cancer-associated driver mutations. Here we review the primary causes of CIN in mitosis and discuss how the oncogenic activation of key signal transduction pathways contributes to the induction of CIN.

## Aneuploidy and CIN in Tumors

Most solid tumors have abnormal chromosome numbers (aneuploidy) and large-scale structural genomic rearrangements (Hanahan and Weinberg, 2000, 2011). Karyotypic analyses show that tumors display both intra- and inter-tumor heterogeneity suggesting that most tumors are not only aneuploid, but also chromosomally unstable. Chromosomal instability (CIN) is defined as a persistent high rate of gain/loss of whole chromosomes (Lengauer et al., 1997). CIN is present in most aneuploid solid tumors and is an important hallmark of genome instability associated with cancer. Since CIN (and the consequent aneuploidy) is caused by aberrant chromosome segregation, it is probable that most cancer cells acquire defects in the machinery responsible for faithful chromosome segregation in mitosis. These defects could arise through either mutation of genes encoding essential mitotic proteins or by imbalances in protein levels or activities that reduce mitotic fidelity (reviewed in Schvartzman et al., 2010; Nicholson and Cimini, 2011).

Aneuploidy and CIN commonly go hand-in-hand because the proximal outcome of CIN is to create deviations in the karyotype. However, it is important to note that aneuploidy and CIN are distinct (Thompson and Compton, 2008). Aneuploidy is a state of abnormal chromosome number and some tumors have abnormal, yet stable karyotypes (i.e., all the cells in the tumor have the same defective karyotype). CIN is a high rate of chromosome mis-segregation that enhances karyotypic diversity in cells within the same tumor, a feature commonly associated to tumor aggressiveness (Storchova and Pellman, 2004; Geigl et al., 2008). On average, CIN cancer cells mis-segregate a chromosome once in every one to five cell divisions (Lengauer et al., 1997; Thompson and Compton, 2008), an event which is thought to drive the genomic re-shuffling that allows cells to acquire new phenotypes such as drug resistance. Significantly, both aneuploidy and CIN have been associated with poor patient prognosis, metastasis, and resistance to chemotherapeutics (Kuukasjarvi et al., 1997; Carter et al., 2006; Walther et al., 2008; Swanton et al., 2009; Bakhoum et al., 2011; Lee et al., 2011; Smid et al., 2011; McGranahan et al., 2012) and recent reports have shown that aneuploidy and CIN play a causal role in tumorigenesis and relapse (Weaver et al., 2007; Baker et al., 2009; Sotillo et al., 2010). Thus, understanding the mechanisms that cause CIN offers an attractive possibility to intervene in tumor aggressiveness and enhance the efficiency of cancer therapy.

Large-scale genome sequencing has revealed the most commonly mutated genes in cancer (Cancer Genome Atlas Research Network, 2008, 2011, 2012; Jones et al., 2008). These cancer-causing genes encode proteins responsible for cell cycle control and cell signaling pathways responsible for cell growth and death. Genome sequencing analyses have revealed very few mutations in genes that encode proteins involved in chromosome segregation during mitosis. Thus, the persistent mis-segregation of chromosomes in CIN tumor cells has been largely attributed to errors arising during mitosis that were not directly linked to the driver mutations in oncogenic signaling pathways. However, the idea that CIN is caused by events that are independent of the oncogenic signaling pathways is beginning to falter. Emerging data show that CIN and the oncogenic signaling pathways responsible for driving tumor formation are closely interrelated. Here we provide a brief overview of the mitotic defects that commonly cause CIN and then explain how oncogenic signaling pathways that are commonly de-regulated in cancer influence mitosis to induce CIN.

## Causes of CIN in Cancer Cells

**Figure 1:**
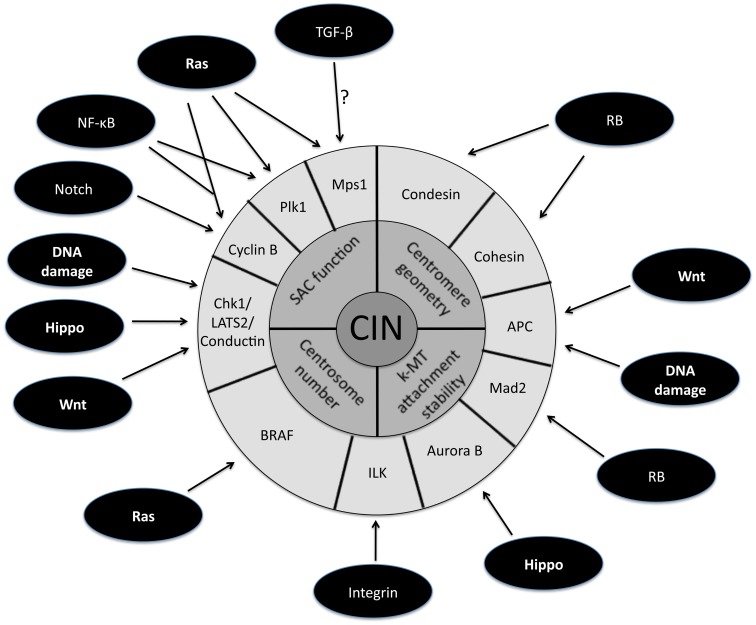
**Schematic representation of the downstream targets of oncogenic signaling pathways that affect mitotic fidelity**. The inner circle represents chromosomal instability (CIN) and the middle circle is composed of the four primary defects in mitosis known to cause CIN. The downstream targets of oncogenic pathways involved in the four primary CIN-causing mitotic defects comprise the outer circle. Note that some downstream targets (e.g., APC) have been demonstrated to play a role in more than one CIN-causing mitotic defect.

The persistently high rate of chromosome mis-segregation associated with CIN in tumor cells has been attributed to four primary defects in mitosis (Figure 1, inner circle). The first mitotic defect proposed to cause CIN was an impaired spindle assembly checkpoint (SAC). This idea stemmed from a study demonstrating that CIN is caused by mutational inactivation of the essential SAC component Bub1 (Cahill et al., 1998). Although the initial identification of mutations in Bub1 was encouraging, subsequent genome sequencing showed that mutational inactivation of SAC components is quite rare (Barber et al., 2008). Moreover, complete loss of SAC function is lethal (reviewed in Kops et al., 2004; Kops et al., 2005). Also, follow up experiments demonstrated that most aneuploid cancer cells possess a functional SAC (Tighe et al., 2001; Gascoigne and Taylor, 2008). These results gave rise to the alternative hypothesis that partial loss of SAC function is responsible for causing CIN. Evidence in favor of this view is derived from the high incidence of aneuploidy and tumorigenesis in mice engineered to have weakened SAC activity (Michel et al., 2001; Dai et al., 2004). Moreover, in humans, reduced SAC activity has been observed in individuals with Mosaic Variegated Aneuploidy (MVA), an extremely rare disease strongly linked to mutations in SAC component BubR1 (Hanks et al., 2004; Suijkerbuijk et al., 2010).

The second mitotic defect shown to cause CIN is the persistence of errors in kinetochore-microtubule (k-MT) attachments as revealed by live imaging of cancer cells (Thompson and Compton, 2008; Compton, 2011). Faithful chromosome segregation relies on the bi-oriented attachment of chromosomes to spindle microtubules. Kinetochores in human cells bind approximately 25 microtubules and errors in the orientation of k-MT attachments arise through the stochastic nature of interactions between microtubules and kinetochores. Merotely is a specific k-MT attachment error defined by single kinetochores that simultaneously attach microtubules oriented toward both spindle poles. Merotely avoids detection by the SAC since kinetochores attain full occupancy of microtubules (albeit with improper orientation). Thus, if cells enter anaphase with merotelically attached kinetochores, the chromatid attached to both spindle poles will fail to segregate properly and lag near the central spindle as other chromosomes move poleward. This can cause chromosome mis-segregation and lagging chromosomes in anaphase were the most common defect in mitosis observed by live cell imaging in cancer cell lines. Error-free mitosis is promoted by microtubule detachment from kinetochores and the stability of k-MT attachments is regulated through the concerted action of several mitotic kinases, notably Aurora B (Lampson et al., 2004; Pinsky et al., 2006). Direct measurements show that many CIN cancer cells have hyperstable k-MT attachments which undermines their ability to correct errors and leads to high rates of chromosome mis-segregation. Importantly, increasing the detachment rate of k-MT improves error correction and is sufficient to restore faithful chromosome segregation in CIN cancer cells (Bakhoum et al., 2009a,b; Kabeche and Compton, 2012). These results are the first genetic demonstration of the correction of CIN and provide direct causal evidence that impaired regulation of k-MT attachments is an underlying cause of CIN in human cancer cells.

The third mitotic defect leading to CIN is the presence of supernumerary centrosomes. Centrosome duplication is tightly regulated during the cell cycle such that normal cells enter mitosis with only two. Two centrosomes foster bipolar spindle formation, yet it has been well documented that cancer cells frequently enter mitosis with more than two centrosomes leading to multipolar spindles (reviewed in Nigg, 2002; Fukasawa, 2005; Bakhoum and Compton, 2009; Anderhub et al., 2012; Vitre and Cleveland, 2012). Interestingly, the frequency of multipolar spindles is higher in prometaphase than in anaphase indicating that centrosomes cluster to promote bipolar spindle formation prior to anaphase onset (Brinkley, 2001; Quintyne et al., 2005; Basto et al., 2008). Despite these centrosome clustering mechanisms, the presence of extra centrosomes prolongs mitosis by delaying satisfaction of the SAC even in cells with bipolar spindles (Yang et al., 2008) suggesting that not all kinetochores are properly attached to microtubules. In support of this, recent studies have shown that the transient multipolar spindles caused by supernumerary centrosomes increase the propensity of merotelic k-MT attachments and elevate the frequency of chromosome mis-segregation (Ganem et al., 2009; Silkworth et al., 2009). Thus, extra centrosomes increase the rate of formation of k-MT attachment errors leading to CIN.

The fourth mitotic defect causing CIN is tied to centromere geometry. The integrity of centromeric structure ensures that kinetochores are positioned in a back-to-back configuration. This creates a geometric constraint that increases the probability that sister kinetochores of a single chromosome will achieve proper bi-orientated attachments to spindle microtubules (Indjeian and Murray, 2007; Loncarek et al., 2007; Sakuno et al., 2009). It has been shown that defects in pericentromeric cohesion undermine the establishment of proper k-MT attachments (Ng et al., 2009). The consequence is an increased rate of formation of k-MT attachment defects that leads to elevated rates of chromosome mis-segregation.

## How Oncogenic Pathways Induce CIN

A large fraction of tumors have driver mutations in genes encoding components of a handful of conserved oncogenic pathways. Although the specific genes that are mutated within each pathway can differ between tumors from different individuals, the common feature is that most tumors have mutations that cause aberrant signaling in these key signal transduction pathways. For example, sequencing analyses of pancreatic cancers and glioblastomas revealed that a core set of 12 signaling pathways are genetically altered in 67–100% of all tumors (Cancer Genome Atlas Research Network, 2008; Jones et al., 2008). Here we discuss how the oncogenic activation of these signaling pathways contributes to the induction of CIN (Figure 1; Table 1).

**Table 1:** **Table summarizing the CIN-causing mitotic phenotypes observed in different cell types and/or models upon de-regulated oncogenic signaling**.

Pathway	Gene(s)	Mitotic phenotype	Model	Reference
RB	E2F	Chromatin assembly/condensation; chromosome mis-segregation; impaired SAC; stabilization of k-MT attachments	Human fibroblasts (WI-38), MEFs, HCT116, T24, HT1197, SW480, U2OS, IMR-90, RPE1, NIH-3T3	Ren et al. (2002), Hernando et al. (2004), Kabeche and Compton (2012)
	RB	Chromosome condensation; impaired centromere structure; heterochromatin structure; chromosome mis-segregation	MEFs; RPE1; Drosophila neuroblasts	Isaac et al. (2006), Coschi et al. (2010), Manning et al. (2010)
	RB, p107, p130	Chromatid breaks; cohesion defects	MEFs	Manning et al. (2010), van Harn et al. (2010)
Wnt	Conductin/AXIN2 and APC	Premature centrosome separation (Plk1-dependent); Impaired SAC (Plk1-dependent)	MEFs; SW480, U2OS, HCT116, DLD1, HEK293T, HeLa	Hadjihannas et al. (2006), Hadjihannas et al. (2010), Ruan et al. (2012)
	Disheveled 2 (Dvl2)	Regulation of MT-plus-ends (Plk1-dependent); Impaired SAC (Mps1, Bub1, and BubR1-dependent)	HeLaS3, U2OS	Kikuchi et al. (2010)
	GSK3β	Mis-alignment; chromosome mis-segregation; micronuclei formation	HeLa, HEK293T, Drosophila embryos	Wakefield et al. (2003), Bobinnec et al. (2006), Tighe et al. (2007)
	β-catenin	Loss of centrosome separation; monopolar spindle formation; chromosome condensation	HeLa, HEK293T, NIH-3T3, MDCK, U2OS, DlD1, HCT116	Kaplan et al. (2004), Bahmanyar et al. (2008), Davalos et al. (2012)
	APC	Hyper-stabilization of k-MT attachment	Mouse ES cells, Ptk2, HT29, SW480, Caco2, LoVo, HCT116, RKO, HEK293T, HeLa, RPE1	Fodde et al. (2001), Zumbrunn et al. (2001), Green and Kaplan (2003), Green et al. (2005), Draviam et al. (2006), Bakhoum et al. (2009a)
DNA damage	p53	Centrosome function (Aurora A, Plk2, and Plk4-dependent)	Mouse hepatocytes	Kurinna et al. (2013)
	unknown component	Centrosome integrity; multipolarity; chromosome mis-segregation	CHO	Hut et al. (2003)
	Chk1	k-MT stability (Aurora B-dependent); Centrosome amplification; Disrupted SAC function (Mad2, BubR1, and Aurora B-dependent)	DT40, HCT116, HEK293T, U2OS, mouse MECs, HC11, NIH-3T3, HeLa, OVCAR-8, OVCA-432, A2780, OVCAR-5	Bourke et al. (2007), Zachos et al. (2007), Carrassa et al. (2009), Peddibhotla et al. (2009), Chila et al. (2013)
	Chk2/BRCA1	Abnormal mitotic spindle assembly; lagging chromosomes	HCT116, HeLa, BJ-hTERT	Stolz et al. (2010b)
	DNA-PKcs	Abnormal spindle formation	HeLa, AT5BIVA	Shang et al. (2010)
Ras	HRas	Weakened SAC	PCCL3	Knauf et al. (2006)
	KRas	Chromosome mis-segregation	DLD1, HCT116	Luo et al. (2009)
	BRAF	Impaired SAC (Mps1-dependent); supernumerary centrosomes	SK-MEL-5, SK-MEL-28, A375, SBcl2, hTERT-HME	Cui et al. (2010)
Notch	Notch	Mitotic delay (CycB1-dependent)	KS-IMM, KS-Y1	Curry et al. (2007)
TGF-β	Ski	Weakened SAC function; lagging chromosomes/chromosome bridges; micronuclei formation	Primary MEFs, SV-40 immortalized MEFs	Marcelain et al. (2012)
	Smad2/3	Impaired SAC function?	Sw480, HeLa	Zhu et al. (2007)
NF-κB	IκB	Centrosome function (Aurora A-dependent); Mitotic arrest (CycB1 and Plk1-dependent); chromosome mis-segregation	HeLa, COS7, Primary MEFs	Prajapati et al. (2006), Irelan et al. (2007)
Integrin	ILK	Centrosome clustering (TACC3 and ch-TOG-dependent); Hyper-stabilization of k-MT dynamics	BT549, MDA-MB-231, MCF7, MCF10A, 184-hTERT, PC3, DU145, BPH-1, HEK293T, HeLa, IMR-90	Fielding et al. (2008), Fielding et al. (2011), Lim et al. (2013)
Hippo	NDR1/Fry/MST2	Chromosome misalignment	HeLa	Chiba et al. (2009)
	MST1	Centrosome overduplication; Stabilization of k-MT attachments (Aurora B-dependent)	HeLa, U2OS, PT67, COS7, RPE1	Hergovich et al. (2009), Oh et al. (2010)
	LATS2	Centrosome fragmentation; chromosome misalignment; loss of SAC activity; cytokinesis failure	Primary MEFs	Yabuta et al. (2007)
	KIBRA	Defects in mitotic spindle formation; chromosome misalignment	HEK293T, MCF7, HeLa	Zhang et al. (2012)

## The RB Pathway

The retinoblastoma tumor suppressor protein (pRB) is a key regulator of the G1 to S transition during the cell cycle (Burkhart and Sage, 2008). It was one of the first tumor suppressor genes identified and is commonly inactivated in several tumor types (Marshall, 1991). It is common for tumors with inactivating mutations in pRB to be aneuploid with an increased susceptibility to changes in DNA ploidy (Isaac et al., 2006; Mayhew et al., 2007; Srinivasan et al., 2007; Amato et al., 2009a,b), and recent reports have shown that pRB influences mitosis through a function that is independent of its role in cell cycle progression (Sage and Straight, 2010). The E2F transcription factor family proteins are downstream targets that are negatively regulated by pRB. These transcription factors regulate the expression of genes whose products are involved in chromatin assembly/condensation, chromosome segregation, and in the mitotic checkpoint such that loss of pRB increases their transcription (Ren et al., 2002). For example, pRB negatively regulates the transcription of the gene encoding SAC protein Mad2 and loss of pRB signaling leads to overexpression of Mad2 giving rise to aneuploidy and CIN (Hernando et al., 2004). A recent study showed that Mad2 stabilizes k-MT attachment in a function that is distinct from its role in the SAC (Kabeche and Compton, 2012). Overexpression of Mad2 hyper-stabilizes k-MT attachments and increases the rate of formation of k-MT attachment errors leading to CIN.

Loss of pRB also alters chromatin structure. For example, pRB has been shown to interact with the Condensin II complex and participate in mitotic chromosome condensation. Loss of pRB accelerates both loss of heterozygosity in the absence of p53 and the rate of tumor formation (Coschi et al., 2010). Moreover, disruption of pRB in mice has been shown to disrupt pericentric heterochromatin leading to centromere fusions, chromosome mis-segregation, and consequently aneuploidy (Isaac et al., 2006). This effect on centromere integrity was corroborated in a subsequent study showing that loss of pRB, p107, and p130 in mouse embryonic fibroblasts (MEFs) causes a G2 arrest coupled with DNA damage which results in chromatid breaks and cohesion defects in mitotic chromosomes (van Harn et al., 2010). Finally, Manning and co-workers demonstrated that pRB depletion alters the centromeric localization of CAP-D3/condensin II, leading to distorted centromere structure and increased inter-kinetochore distance. These centromere distortions increase the propensity of merotelic k-MT attachment leading to CIN (Manning et al., 2010). Taken together, these studies show that in addition to altered cell cycle regulation, the loss of pRB alters centromere geometry to increase the rate of formation of k-MT attachment errors and hyper-stabilizes k-MTs to reduce the rate of correction of attachment errors (Hernando et al., 2004; Isaac et al., 2006; Manning et al., 2010; van Harn et al., 2010).

## The Wnt Signaling Pathway

The Wnt signaling pathway plays a critical role in stem cell renewal, survival, and differentiation, as well as in the normal development and homeostasis of tissues including the mammary gland (Boras-Granic and Wysolmerski, 2008). De-regulation of Wnt signaling plays a causal role in breast cancer (reviewed in Prosperi and Goss, 2010). Aberrant Wnt signaling has been shown to play role in accelerating tumorigenesis by promoting CIN in the absence of p53 (Donehower et al., 1995) and numerous studies have implied that Wnt signaling is directly involved in mitotic regulation (reviewed in Niehrs and Acebron, 2012).

Excessive Wnt signaling up-regulates expression of conductin/AXIN2, a protein involved in SAC signaling and centrosome cohesion (Hadjihannas and Behrens, 2006; Hadjihannas et al., 2010). Conductin localizes to mitotic spindles and high conductin expression attenuates SAC activity via a Polo-like kinase 1 (Plk1)-dependent mechanism (Hadjihannas et al., 2006). Additionally, Axin phosphorylation by Plk1 is essential for proper centrosome function (Ruan et al., 2012). Plk1 also phosphorylates Disheveled 2 (Dvl2), a central component of the Wnt signaling pathway that plays a role in SAC activation by recruiting SAC components Mps1, Bub1, and BubR1 to kinetochores (Kikuchi et al., 2010). In addition to conductin and Dvl2, both GSK3β and β-catenin have been shown to be involved in centrosome separation and to regulate spindle microtubules during mitosis (Wakefield et al., 2003; Kaplan et al., 2004; Bobinnec et al., 2006; Bahmanyar et al., 2008). Consistently, treating human cells with GSK3β-inhibitors was shown to induce CIN (Tighe et al., 2007). Wnt signaling also regulates mitosis through the conserved protein Adenomatous Polyposis Coli (APC). APC enhances the correction of k-MT attachment errors because truncation or depletion of APC in human cells increases k-MT stability through interactions with microtubule plus-ends and induces chromosome mis-segregation (Fodde et al., 2001; Zumbrunn et al., 2001; Green and Kaplan, 2003; Green et al., 2005; Draviam et al., 2006; Bakhoum et al., 2009a). However, since APC is commonly mutated and/or truncated in tumor cells, the role of APC in regulating microtubule plus-ends is most likely independent of Wnt signaling. Finally, SMC2, a core subunit of the condensin complex, is a transcriptional target of the Wnt signaling pathway demonstrating a functional link between Wnt signaling and chromosome condensation (Davalos et al., 2012). These results suggest that excessive Wnt signaling decreases the efficiency of the correction of k-MT attachment errors and increases the rate of formation of k-MT attachment errors by stabilizing k-MT attachments, influencing centrosome cohesion, and affecting centromere geometry.

## The Ras Signaling Pathway

The Ras oncogene is one of the most commonly mutated genes across an array of cancer types and de-regulated signaling through receptor-tyrosine kinase pathways caused by mutant Ras (or its GAP or GEF) affects cell cycle regulation, apoptosis, and cell survival (reviewed in Malumbres and Barbacid, 2003). Apart from the well-characterized roles that Ras signaling plays in cell cycle progression, it has also been implicated in the maintenance of genome integrity and this hypothesis gains strength with several important observations (reviewed in Kamata and Pritchard, 2011). First, cells harboring gain-of-function Ras mutations exhibit changes in DNA ploidy and genomic rearrangements in mouse models and rat cells (Ichikawa et al., 1990; Denko et al., 1994; Saavedra et al., 2000; Baker et al., 2013). Consistently, oncogenic HRas^G12V^ expression in rat thyroid cells was shown to weaken the SAC response to microtubule targeting drugs (Knauf et al., 2006). Furthermore, expression of oncogenic Ras at endogenous levels is sufficient to generate chromosome mis-segregation in cancer cells perhaps through Plk1 and the Anaphase-Promoting Complex/Cyclosome (APC/C) (Luo et al., 2009). Importantly, an elegant set of experiments using primary MEFs grown under conditions that preserve diploid karyotypes showed that expression of HRas^G12V^ resulted in the induction of CIN, an outcome that was enhanced by the absence of p53 (Woo and Poon, 2004).

The ERK pathway is the best-characterized signaling pathway downstream of Ras and its contribution to mitotic progression in human cells appears to be cell-type dependent. For example, depletion of ERK1/2 in keratinocytes, but not in fibroblasts, induces a CyclinB1-dependent G2/M arrest (Dumesic et al., 2009). Additionally, ERK1/2 has been detected at centrosomes and kinetochores during mitosis (Shapiro et al., 1998; Lou et al., 2004) but a functional role for ERKs during mitosis has so far remained elusive. Furthermore, another downstream target of Ras is Raf kinase, and human melanoma cells expressing oncogenic B-RAF^V600E^ were shown to “hyperactivate” the SAC through stabilization of Mps1 and induce supernumerary centrosomes resulting in chromosome mis-segregation accompanied by aneuploidy (Cui et al., 2010). These reports provide evidence that deranged Ras signaling can induce CIN although the precise molecular mechanism leading to elevated chromosome mis-segregation remain elusive.

## The Transforming Growth Factor beta (TGF-β) Signaling Pathway

TGF-β signaling is involved in the regulation of cell proliferation, differentiation, motility, adhesion, and apoptosis and therefore de-regulation of the TGF-β pathway is thought to play a critical function in cancer development. However, the role of TGF-β signaling in tumorigenesis is somewhat paradoxical since it acts as a tumor suppressor at early stages of cancer development and as an oncogene at terminal stages of the disease by initiating a transcriptional program required for the activation of genes involved in cell metastasis and invasion (reviewed in Massague, 2008; Barcellos-Hoff and Akhurst, 2009; Padua and Massague, 2009). The primary intracellular mediators of TGF-β are the Smad proteins, which are phosphorylated and activated by the TGF-β Receptor I (TβRI) kinase. This phosphorylation event promotes translocation of Smad proteins to the nucleus where they cooperate with transcription factors to regulate gene expression (Derynck and Zhang, 2003).

The link between TGF-β signaling and mitosis came from the observation that endogenous Smads bind microtubules and that microtubule-depolymerization in cells triggers phosphorylation of Smad2 (Dong et al., 2000). Subsequently, it was shown that the conserved SAC protein Mps1 co-purifies with the Smad protein complex and that nocodazole treatment results in Mps1-dependent phosphorylation and consequent activation of Smad2/3 proteins (Zhu et al., 2007). Interestingly, regulation of Smad3 phosphorylation in mitosis was not only dependent on Mps1, but also on ERK activation (Hirschhorn et al., 2012). This raises the possibility of cross-talk between TGF-β activation and Ras signaling in mitosis. Another link between TGF-β signaling and mitosis involves the transcriptional co-activator protein Ski, which acts as a negative regulator of TGF-β signaling. Ski protein levels peak during mitosis and immunofluorescence imaging of U2OS cells demonstrates that Ski localizes at centrosomes and mitotic spindles (Marcelain and Hayman, 2005) and is directly phosphorylated by Aurora A *in vitro* (Mosquera et al., 2011). The functional significance of this localization became clear through a study demonstrating that Ski−/− MEFs have a weakened SAC and show increased rates of lagging chromosomes during anaphase resulting in micronuclei formation and the generation of aneuploidy and CIN (Marcelain et al., 2012). Thus, the activities of Smads and Ski show that alterations in TGF-β signaling disrupt chromosome segregation during mitosis to promote CIN.

## The Nuclear Factor Kappa-Light-Chain Enhancer of Activated B Cells (NF-κB) Pathway

The connection between inflammation and cancer was described several decades ago and this connection has been strengthened by the identification of constitutively active NF-κB signaling in many cancer types (Karin and Greten, 2005; Maeda and Omata, 2008). Induction of this pathway by specific stimuli leads to phosphorylation of the IκB complex resulting in its targeted destruction and activation of NF-κB. NF-κB is then imported in the nucleus where it activates transcription of target genes involved in immune responses and inflammation.

The IκB complex is composed of two catalytic kinase subunits: IKKα and IKKβ and both kinases have been shown to play distinct roles in mitotic fidelity. Depletion of IKKα in HeLa cells induces a mitotic arrest caused by increased Cyclin B and Plk1. IKKα was also shown to directly phosphorylate and modulate Aurora A kinase activity at centrosomes (Prajapati et al., 2006). These effects appear to be IKKβ-independent, however perturbation of IKKβ in HeLa cells promotes multipolar spindle formation, chromosome mis-segregation and de-regulation of Aurora A stability. This suggests that disruption of IKKα and IKKβ converge on Aurora A kinase (Irelan et al., 2007).

Additional involvement of this pathway in mitosis is through signal adapter proteins. The signaling adaptor p62 is necessary for Ras to trigger IκB and consequently activate the NF-κB pathway. It has also been implicated in mitotic regulation (Moscat and Diaz-Meco, 2012). A recent report demonstrated that Cdk1 phosphorylates p62 to regulate Cyclin B levels during mitosis suggesting that p62 may play a role in SAC maintenance (Linares et al., 2011). Importantly, the authors demonstrate that expression of a non-phosphorylatable p62 increases lagging chromosome rates during anaphase and induces micronuclei formation, features which are consistent with the induction of CIN.

## Integrin Signaling and Cell Adhesion

One important characteristic of transformed cells is their ability to grow in an anchorage-independent fashion and this feature of cancer cells has led to the suggestion that Integrin signaling may play an important role in tumorigenesis. Integrin signaling is thought to couple proliferation and survival signaling with anchorage-dependent growth. De-regulated Integrin signaling is thought to provide a metastatic advantage for tumor cells (reviewed in Giancotti and Ruoslahti, 1999).

A key regulator of Integrin signaling is Integrin-linked kinase (ILK). ILK was identified as an interactor of β1- and β3-integrin subunits which localizes to both focal adhesions and centrosomes and the cellular functions of ILK include cytoskeleton organization, cell adhesion, and migration (reviewed in Hannigan et al., 2011). ILK levels have been found to be elevated in many cancer types and correlated to poor patient prognosis (McDonald et al., 2008). Recently, ILK was reported to play an important role in centrosome clustering in cancer cell lines by modulating the microtubule associated proteins TACC3 and ch-TOG (Fielding et al., 2008, 2011). Centrosome de-clustering induces chromosome mis-segregation providing a role for ILKs in mitosis. A more recent study demonstrated that pharmacological inhibition of ILK causes hyper-stabilization of k-MT dynamics resulting in increased centromeric tension in aligned chromosomes (Lim et al., 2013). Stabilization of k-MT attachments is a proven mechanism of CIN in cancer cells (Bakhoum et al., 2009a,b) indicating that ILK is involved in the regulation of proper chromosome segregation and may contribute to CIN.

## The Hippo Signaling Pathway

The Hippo pathway was originally identified in Drosophila and is conserved in mammals. This pathway is responsible for coordinating cell proliferation and apoptosis to govern mechanisms of cell contact inhibition, organ size control, and cancer progression (reviewed in Saucedo and Edgar, 2007). In agreement, this pathway contains both oncogenes and tumor suppressor genes whose homologs have been identified in mammals (Overholtzer et al., 2006; Zender et al., 2006; Harvey and Tapon, 2007).

The first clue that the Hippo signaling pathway could be directly contributing to mitotic progression came from a study reporting that the activity of MST1 and MST2, two essential kinases of the Hippo pathway in mammalian cells, increases during mitosis, and that this effect was enhanced by microtubule-depolymerizing drugs (Praskova et al., 2008). A subsequent study reported that depletion of NDR1, a downstream target of MST kinases, causes MST2-dependent mitotic chromosome misalignment in HeLa cells (Chiba et al., 2009). Independently, NDR phosphorylation by MST1 was also demonstrated to play an important role in the control of centrosome duplication (Hergovich et al., 2009). Similarly to MST2, MST1 was also reported to participate in chromosome alignment by directly phosphorylating Aurora B to limit its kinase activity and promote stable k-MT attachments during mitosis, demonstrating a functional link between Hippo signaling and the correction of k-MT attachment errors (Oh et al., 2010).

Another link between the Hippo pathway and mitosis is through the large tumor suppressor 2 (Lats2). Lats2 is a kinase involved in Hippo signaling which has been shown to inhibit Cdk1 activity in HeLa cells (Kamikubo et al., 2003). Interestingly, Lats2-deficient MEFs show severe mitotic defects including centrosome fragmentation, chromosome misalignment, loss of SAC activity, and consequent cytokinesis failure (Yabuta et al., 2007). Furthermore, Lats2 localizes to centrosomes in mitosis and its localization is dependent on phosphorylation by Aurora A (Toji et al., 2004). Finally, the WW domain-containing protein KIBRA (enriched in KIdney and BRAin) was recently identified as a novel regulator of the Hippo pathway and was shown to be phosphorylated by Aurora A, Aurora B (Xiao et al., 2011b), and Lats2 (Xiao et al., 2011a). It was subsequently shown that depletion of KIBRA causes defects in mitotic spindle formation and chromosome alignment suggesting that it is an active player in mitotic fidelity (Zhang et al., 2012). Collectively, these reports show strong evidence that Hippo signaling plays an important role in generating CIN by affecting faithful chromosome segregation during mitosis although many of the molecular mechanisms involving Lats2 and KIBRA in chromosome segregation are currently unclear.

## The DNA Damage Response

The DNA damage checkpoint is a conserved molecular mechanism essential for DNA repair and has been commonly associated with the maintenance of genome stability and carcinogenesis (Hoeijmakers, 2001). Intriguingly, this checkpoint responds to DNA damage throughout the cell cycle, except in mitosis. The role of the DNA damage pathway in affecting mitotic fidelity is currently a topic of intense investigation (reviewed in Hayashi and Karlseder, 2013). One area focuses on the role of p53, which in principle contributes to CIN through two mechanisms. One relates to how the DNA damage pathway responds to chromosome mis-segregation. Loss of p53 function has been shown to provide tolerance for aneuploidy and further propagation of those aneuploid cells can induce CIN (Bunz et al., 2002; Thompson and Compton, 2010). The second relates to how p53 regulates expression of genes that encode proteins involved in mitosis such as Aurora A, Plk2, and Plk4 (Kurinna et al., 2013). The stabilization of p53 in response to DNA damage increases the expression of these genes hinting at a more direct role in disrupting faithful chromosome segregation.

Numerous studies have implicated DNA damage pathway components other than p53 in the induction of CIN by tampering with the fidelity of chromosome segregation during mitosis. In mammalian cells, centrosome integrity was shown to be affected upon DNA damage, resulting in multipolarity and consequent chromosome mis-segregation (Hut et al., 2003). Chk1, a component of the DNA damage and replication checkpoints, has been directly implicated in mitotic regulation through several independent mechanisms. First, it is required for centrosome amplification upon DNA damage (Bourke et al., 2007). Second, it participates in the k-MT error correction machinery by directly phosphorylating and enhancing Aurora B activity *in vitro* (Zachos et al., 2007). Third, Chk1 depletion in human osteosarcoma cells disrupts the SAC by affecting Mad2 and BubR1 levels and consequently increases the rate of chromosome mis-segregation (Carrassa et al., 2009; Peddibhotla et al., 2009). Finally, a recent report has demonstrated that apart from its role in phosphorylating Aurora B, Chk1 also directly phosphorylates SAC component Mad2 suggesting multiple levels of mitotic regulation by Chk1 (Chila et al., 2013).

Depletion of Chk2 or abrogation of its kinase activity was shown to cause mitotic delay, promote the generation of lagging chromosomes and consequently induce CIN through a BRCA1-dependent but p53-independent mechanism (Stolz et al., 2010a,b). Importantly, these reports demonstrate that the DNA damage pathway combines tumor-suppressive properties with the maintenance of chromosome integrity (Sato et al., 2010). Chk2, together with Plk1, Cdk1, and 53BP1 (p53-binding protein-1) was also postulated to participate in a mitotic phosphorylation feedback network responsible for inactivating the G2/M DNA damage checkpoint (van Vugt et al., 2010). In budding yeast, the Chk2 homolog Rad53, also plays an important role in suppressing the cleavage of sister chromatid cohesion and spindle elongation to preserve genomic stability (Zhang et al., 2009).

In addition, DNA-dependent protein kinase catalytic subunits (DNA-PKcs), known for their active role in DNA non-homologous end joining (NHEJ), have also been shown to localize to centrosomes, kinetochores, and the midbody (Shang et al., 2010). However, the role that DNA-PKcs play in mitotic fidelity is currently unclear. Importantly, more than 50% of all cancers carry mutations in components of the DNA damage pathway (Hollstein et al., 1991) and these lines of evidence strongly point to the participation of this pathway in the formation or correction of k-MT attachment errors which are known to be a common cause of CIN.

Despite the compelling evidence that CIN is caused by defective chromosome segregation in mitosis (Cimini et al., 2001; Loncarek et al., 2007; Thompson and Compton, 2008; Bakhoum et al., 2009a; Ganem et al., 2009; Silkworth et al., 2009; Manning et al., 2010; Thompson et al., 2010; Nicholson and Cimini, 2011; Schvartzman et al., 2011; Hood et al., 2012; Kabeche and Compton, 2012; Vitre and Cleveland, 2012), a recent report suggests that CIN is caused by pre-mitotic events induced by replication stress (Burrell et al., 2013). The authors state that most anaphase defects arise through pre-mitotic defects suggesting that activation of DNA damage through replication stress reflects a cause, rather than a consequence, of segregation errors (Burrell et al., 2013). The expected outcome of replication stress is chromosome fragments, including those that are acentric, which was documented in that work. So, that is not surprising. However, the surprise is the proposition that replication stress causes whole chromosome mis-segregation through a mechanism independent of defects of the segregation process in mitosis. DNA replication stress has been observed across several tumor types (Dereli-Oz et al., 2011) and the recent results illustrate a need to search for the mechanism that causes whole chromosome mis-segregation as a consequence of replication stress.

Interestingly, recent work has shown that chromosome segregation defects can lead to DNA damage. For example, it was shown that lagging chromosomes in anaphase tend to be trapped in the cytokinetic furrow resulting in DNA double strand breaks (Hoffelder et al., 2004; Janssen et al., 2011). Also, lagging chromosomes tend to form micronuclei in the subsequent G1 phase. The chromosomes trapped in micronuclei do not replicate on time with the major nucleus and are occasionally pulverized in the subsequent mitosis (Crasta et al., 2012). These results reveal a potentially vicious cycle. Chromosome segregation errors lead to DNA damage and that damage may promote further chromosome mis-segregation. Analyzing the link between oncogenic signaling and DNA replication might therefore provide important clues regarding the induction of CIN via pre-mitotic events.

## Signaling Pathway Cross-Talk

This summary provides convincing evidence for the role of commonly mutated oncogenic signaling pathways in cancer for the induction of CIN. This summary has specifically treated each signaling pathway as an independent linear entity. However, in actuality, there is extensive interconnection between signaling pathways leading to cell-, tissue-, and cancer-type specificity in effects and outcomes of pathway activation. In particular, the specific experimental conditions used to test the role of a signaling pathway could impose a great degree of variability on the results generated and interpretations need to be made carefully based upon the cell, tissue, or cancer model system being employed.

For example, Notch signaling was first linked to tumorigenesis through the identification of a frequent chromosomal translocation found in a subset of human T-cell acute lymphoblastic leukemias (T-ALL) (Ellisen et al., 1991). It has since been shown to be involved in several tumor types (reviewed in Allenspach et al., 2002). Interestingly, Notch can behave as both a dominant oncoprotein and as a tumor suppressor which is not surprising given the diversity of Notch functions reported to date (Lobry et al., 2011). Although Notch mutations have been associated with poor patient prognosis, no clear connections to CIN have thus far been established. However, Notch signaling is connected to most other signaling pathways. Expression of Wnt activates Notch signaling in human mammary epithelial cells and this link has been confirmed in a panel of 34 breast carcinomas suggesting that there is a need for Notch-Wnt cross-talk during mammary tumorigenesis (Ayyanan et al., 2006). TGF-β signaling has also been demonstrated to up-regulate Notch in multiple types of mammalian cells and both TGF-β and Notch can synergistically regulate the same target genes in many cell types (Blokzijl et al., 2003). Notch ligand JAG2 is induced by Hedgehog signaling during carcinogenesis (Katoh, 2007) and the cross-talk between Ras and Notch pathways has been amply documented in several tumor types including adenocarcinomas, gliomas, leukemias, and breast cancers (Chiang et al., 2008; Kindler et al., 2008; Mittal et al., 2009; Hanlon et al., 2010; Xu et al., 2010). Aberrant Notch signaling can also increase NF-κB activity by directly interacting with NF-κB and promoting its nuclear retention (Shin et al., 2006). Furthermore, upstream regulators have been shown to combine the promotion of constitutive Notch signaling with an attenuation of the DNA damage pathway (Colaluca et al., 2008) providing evidence to suggest that activation of these pathways via different stimuli may differentially affect how these pathways operate. Thus, Notch signaling may be an important conspirator with these other signaling pathways to affect cell cycle regulation and/or induce CIN.

Many of these signaling pathways also interconnect with the DNA damage response. For example, the RB pathway has also been shown to cooperate with TGF-β signaling in the context of mammary gland development (Francis et al., 2009) and a molecular link between RB signaling, the DNA damage checkpoint and the SAC has recently been reported (Jahn et al., 2013). Cross-talk between the DNA damage pathway and the Hippo pathway via LATS2 has also been established in liver cells (Kurinna et al., 2013). In addition, NF-κB signaling has been shown to cooperate with Ras signaling through the adaptor protein p62 (Linares et al., 2011).

Since cross-talk between pathways is dependent upon intricate feedback loops and intersections at critical nodes that ensure cellular homeostasis it is important to understand the cell-, tissue-, and cancer-specific context in how these pathways interact. For example, during breast cancer progression, TGF-β represses NF-κB in normal cells but activates NF-κB in malignant counterparts (Neil and Schiemann, 2008). A cautionary note is that many of the results summarized here were obtained using cancer cell lines in which the genetic background is often unknown which may confound clear interpretation. The interconnectedness of cell signaling circuitry adds a layer of complexity that challenges our understanding of how signaling pathways not only drive tumorigenesis but induce CIN.

## Conclusion

The oncogenic signaling pathways described here all play dual roles. They act as drivers for tumorigenesis and they induce CIN. This connection to CIN arises because they disrupt the careful orchestration of events required for accurate chromosome segregation during mitosis by decreasing the rate of correction of k-MT attachment errors and/or increasing the rate of formation of those errors through extra centrosomes or disruption of centromere geometry. The notion that tumor suppressor genes (and, by extension, oncogenes) combine their known roles in cell cycle progression, growth, and differentiation with the induction of genomic instability is not necessarily new and a substantial body of evidence supports this (reviewed in Coschi and Dick, 2012). The central point we are making here is that the molecular connections between these signaling pathways and CIN are becoming clearer as insights into the underlying mechanisms generating CIN are married to our understanding of these signaling pathways. Collectively, these reports demonstrate that there is tight communication between mitosis and oncogenic signaling suggesting that mutations leading to mis-regulation of oncogenic pathways not only cause aberrant cell cycle regulation, but also modulate mitosis to generate CIN.

The integration of oncogenic signaling pathways with the induction of CIN changes the way we think about these processes. They can no longer be considered as separate cancer-associated insults. The persistent chromosome mis-segregation in CIN cancer cells provides an agent of genomic change that permits new phenotypes to emerge such as resistance to the toxicity imposed by chemotherapeutic agents. As such, the oncogenic signaling pathways that combine the promotion of cell cycle progression with the induction of CIN may be the most difficult to treat offering some insight into the correlation of CIN and poor patient prognosis. The unanswered question is whether the targeted inhibition of the cancer driving signaling pathway would also rob the cancer cell of its ability to adapt through CIN. Or, once initiated, does CIN become a self-sustaining process that can’t be reversed even if the activity from the oncogenic pathway that started it is quelled? Also, why have signaling pathways evolved these dual roles to influence mitotic events? Is their role in mitosis a part of their normal function that becomes exaggerated upon oncogenic activation or is their mitotic role an off-target effect resulting from a dereliction of function after oncogenic activation? Understandably, these dual roles may be of particular value for stable diploid cells where it is important to stall cell cycle progression following chromosome mis-segregation. However, in the context of tumorigenesis this may turn out to be a double-edged sword that combines de-regulated cell cycle progression with the disruption of mitosis to generate the highly complex karyotypes typical of solid tumors. Our understanding of the links between oncogenic pathways and CIN provide the tools to begin to answer these important questions.

## Conflict of Interest Statement

The authors declare that the research was conducted in the absence of any commercial or financial relationships that could be construed as a potential conflict of interest.
